# Law and psychiatry—current and future perspectives

**DOI:** 10.3389/fpubh.2022.968168

**Published:** 2022-08-24

**Authors:** Tilman Steinert, Tanja Henking

**Affiliations:** ^1^Clinic for Psychiatry and Psychotherapy I, Ulm University, Ulm, Germany; ^2^Centres for Psychiatry Suedwuerttemberg, Ravensburg, Germany; ^3^Department of Psychiatry, Tuebingen University, Tuebingen, Germany; ^4^University of Applied Sciences Würzburg-Schweinfurt, Wuerzburg, Germany

**Keywords:** psychiatry, law, legislation, patient, patients' rights

## Abstract

We describe relevant interfaces between law and psychiatry and current ethical and legal views and changes within the past decades. Ideas of patient autonomy and patients' rights have been major drivers of changes in legal frameworks. We describe developments in the areas of patient information and informed consent, involuntary placement and involuntary treatment, use of coercive measures, forensic psychiatry, digital mental health, data privacy, physician liability, suicide, assisted suicide, euthanasia, end of life decision-making, advance directives, legal and illegal drugs, and delegation and substitution of professional activities. There is no unidirectional pathway between law and ethics. Views, conflicts, and requirements differ between countries and within countries and will need to be balanced according to the societies' changing values also in the future.

## Law and psychiatry: Interfaces

Psychiatry and law have many interfaces. Perhaps the classic example in the public's mind is the appearance of the psychiatric expert in the criminal court. However, the interfaces are much more numerous than this suggests and have increased over the years. While psychiatrists resisted any interference by the judicial system in their supposed sole competence until the middle of the 20th century, their profession, as in other areas of medicine, has since been shaped more and more by legal issues. Patients' rights apply equally to those with mental illness. The wider recognition of the fact has impacted treatment. Some of the challenges faced by psychiatry are similar to those in medicine in general and include questions regarding information disclosure, informed consent, data privacy, and liability in the event of treatment failures. A feature of psychiatry that is not unique to this specialty of medicine but gains most public attention is that it treats people who may temporarily or permanently lack the capacity for free decision-making. The necessity to make decisions on behalf of these people in an appropriate manner to protect their and others' rights makes them highly vulnerable to disrespectful or abusive practices. Thus, a central objective of legislation is to prevent misuse and to protect the rights of particularly vulnerable persons. Ideas around rights, autonomy, and the duty to protect have become central to the regulation of the profession. The tension between freedom and a duty of care, as well as self-determination and protection, has to be explored again and again. We will outline briefly the most relevant areas where legal frameworks follow the evolution of societal and ethical views on mental illness and psychiatric institutions. While many of these legal issues were first developed in high-income countries, they now encompass low- and middle-income countries (LMICS) as well.

## Patient information and informed consent

Informed consent is at the heart of today's understanding of patient autonomy in psychiatry in particular and medicine in general. In recent years, the requirement to provide information and documentation has expanded considerably in most countries. In the treatment of patients, the requirement for information and informed consent has been increasingly regulated by law. The duty to inform patients about the benefits and risks of treatment in easy-to-understand language extends not only to invasive therapies but also to medication and even psychotherapy (1). The legal obligation to provide patients with comprehensive, evidence-based information has raised a series of ethical questions that need careful discussion. E.g., can full disclosure of diagnoses (e.g., schizophrenia, personality disorder) lead to stigmatization? Should patients be told about limited treatment expectations (e.g., regarding antidepressants), or does this conflict with the physician's duty to offer hope (2) A question that is of relevance in medicine in general, does perfect information disclosure annihilate any positive placebo effect that contribute considerably to positive outcomes? On the other hand, insufficient information and education can limit a patient's competence to deal with their condition and lead to non-adherence with treatment. A particular challenge is the assessment of capacity to consent that can change over time in an individual patient, resulting in the need for substituted or assisted decision-making where appropriate. From the legal perspective, assessment of capacity—as a legal construct—must be a dichotomous decision (present or not). From the psychiatric perspective, however, capacity must be understood as a dimensional phenomenon. The translation of these different perspectives is a continuous challenge for all parties involved.

## Involuntary placement and treatment

The legal framework for involuntary placement and treatment is a subject of discussion from the perspective of the UN-CRPD. Traditional mental health laws are based on the criteria of disorders and dangerousness (to self or others), thus discriminating against people with mental illnesses compared to those with somatic disorders and those who are deemed dangerous without having a mental disorder. One pathway for future legislation could be a capacity-based law that would apply to people with both mental and somatic disorders (3). Certain countries have begun to move in this direction, excluding involuntary hospital admission and treatment for people with intact decision-making capabilities irrespective of their diagnosis (4). Furthermore, until the beginning of the century (though in some countries this still applies), the prevailing idea was that involuntary placement and treatment were more or less the same because treatment would take place anyway if a person was admitted involuntarily to a hospital. From a legal perspective, this is not the case, as treatment can be carried out without hospital admission (in the form of an involuntary outpatient commitment) and vice versa (i.e., hospitalization without treatment). Opinions differ as to which of these measures are less intrusive from the perspective of human rights and medical ethics. In any case, from a legal point of view, depriving people of their liberty and treatment against their will are distinct because different rights are violated. The principle of proportionality, in particular the question of the “*mildest mean*s,” is decisive when individual cases are being assessed. During the past decade, these separate perspectives have been incorporated into the law in some countries. From a psychiatric perspective, it is absolutely necessary to avoid developments that can result in long-term detention of people with psychotic disorders in psychiatric facilities without possibilities to treat them. This applies for civil commitment and forensic psychiatry as well.

Finally, community treatment orders (CTOs), encompassing different types of compulsory outpatient treatment, are another subject of debate at the psychiatry-law interface. They have been introduced in many countries, though the manner of use varies widely. As yet, there is no convincing evidence of their effectiveness (5).

## Coercive measures

Seclusion and physical, mechanical, and chemical restraint (which is not the same as involuntary treatment) are the most intrusive interventions against patient autonomy. Formerly regarded as inevitable responses in a part of admitted psychiatric patients, awareness has increased to the point where these measures are regarded as considerable infringements of human rights. Consequently, clear legal rulings are necessary as to their admissibility, their proportionality to the danger to be adverted, the need to choose the mildest measure in each case, and the requirements of supervision, debriefing, and documentation. Most far-reaching worldwide, Germany has introduced the requirement of a judge's decision at the patient's bedside in every instance of mechanical restraint that lasts more than 30 min. Constant 1:1 care is also obligatory in these situations (6). Several countries in Europe and Northern America have legally implemented registries for coercive measures. Such registries are important for many aspects of epidemiological knowledge, quality management, and cross-sectional and longitudinal evaluations.

## Forensic psychiatry

Forensic psychiatry encompasses at least three highly relevant legally regulated areas: (i) admission (diminished or lack of criminal responsibility and dangerousness, type of mental disorder to be ascertained, seriousness of the offense); (ii) stay (patients' rights, proportionality of length of stay, quality of treatment offered); and (iii) discharge (prerequisites and post-discharge treatment supervision). The above-mentioned principles of patient autonomy are having an ever-greater impact on the legal framework for forensic psychiatry, notwithstanding the tension that exists between the need for safety and patients' rights. Forensic psychiatry is severely concerned by financial restrictions and reduced availability of staff on the labor market in many countries. This raises the question of an appropriate treatment offer. Appropriate buildings and staffing will be absolutely necessary to justify its existence in the future.

## Digital mental health

Digital mental health is regarded as the most promising field for innovations in mental health care, particularly for LMICs. Vast numbers of digital tools for (psycho-) therapeutic interventions have become available for many indications, but the question is whether these are products should be offered in a competitive free market to everyone for a relatively low cost or whether they are specific health interventions for specific indications and should therefore be prescribed by physicians and financed by health insurance companies or national health services. In LMICs, digital tools can provide mental health care for people who would otherwise have no access to services. In high-income countries, legal rulings are increasingly focused on evidence-based quality, data privacy, and reimbursement for digital interventions (7).

Digital phenotyping offers even more potential. This is a smartphone-based technology that uses emotional momentary assessments (actively provided by the patient), as well as passive data (e.g., geo-data and meta-data such as finger pressure and velocity during smartphone use), and, possibly, data from text messages (8). For many LMICs or people without sufficient health insurance in high income countries such as the United States, this might be the only mental health facility available to the public in the future. However, the regulation and transparency of data ownership and use and information disclosure will require careful monitoring. There is an increasing discrepancy between well-designed research projects that are thoroughly reviewed by universities' ethics committees and in transparent developments conducted by big digital companies and authoritarian states that go far beyond research projects both in intention and financial power. Digital health technologies challenge legislation in multiple ways, some of which are no doubt yet to be discovered (9).

## Data privacy and data sovereignty

Data privacy has become an issue of increasing importance not only with respect to digital technologies but also to patient files and research data. While patient files were previously considered to be the documenting physician's personal property, the prevailing view nowadays is that patients should have access to any information associated with their illness, with some exceptions relating to the rights of any third parties who are mentioned. The “*open notes*” approach, which appeared in some European countries and the United States only about a decade ago, is now widely considered to be a non-problematic ethical and legal requirement. The concept can be developed further possibly with records in patient files completely being accessible for patients not only retrospectively but at any time, reflecting a non-paternalistic concept of the patient–physician relationship (10). In contrast to these desirable developments from the patient's perspective, the biggest threat for individual mental health data is the access to smartphone data by digital companies and governmental institutions in some States (and will probably be much more in the future).

## Suicide, assisted suicide, and euthanasia

Another facet of the changing views of patient autonomy in modern societies is the issue of suicide, assisted suicide, and euthanasia. Probably with no other issue do attitudes, values, and legislation differ so much, both between and within countries. Solutions to the conflict between the State's duty to protect life and the individual's right to dispose of one's own life vary widely and both latitudinally and longitudinally. Lines of conflict are the impunity of suicide in the first instance, assistance to suicide in the second, and euthanasia in the third. The distinction between suicide, and assisted suicide on the one hand, and euthanasia as the killing of a human being on the other is an ethically and legally significant boundary. Some countries (including Belgium, the Netherlands, New Zealand, Canada, and Spain) have introduced very liberal legislation, with many ethical discussions ongoing. Their laws often focus on people with somatic, life-limiting illness. Other countries still take very restrictive positions, particularly where religion still plays an influential role in politics.

People with mental disorders who can lack decision-making capacity for far-reaching decisions with fatal outcomes deserve special attention; at the same time, and according to the UN-CRPD, they should have the same rights as others and should therefore not be discriminated against. A unique and very challenging requirement for psychiatrists is to assess whether the will to die is or is not a symptom of a mental disorder.

Other ethically complex questions concern the right of patients in forensic hospitals or prisoners to commit suicide. There is a particular tension between respecting the autonomy of such particularly vulnerable people and protecting them. The question of liability is also an important consideration. Uncertainty about liability in cases of patient suicide is a typical severe concern of psychiatrists worldwide, and it encourages paternalistic attitudes. However, maintaining a balance between protecting the patient and not violating their rights remains a fundamental challenge in psychiatry in all countries where court decisions reflect prevailing and changing views. Guidelines and evidence-based recommendations can help in such instances.

## End of life decision-making and advance directives

Advance directives have become an important tool for determining one's will in the case of life-limiting illnesses, particularly where the individual lacks mental capacity as a result of delirium, dementia, or coma. Their binding nature, scope, and interpretation should be governed by statutory provisions. This has been realized in most high-income countries, and it is a topic of interest in many LMICs (11). For people whose mental illnesses may impair decision-making capacity in times of acute crisis, psychiatric advance directives and joint crisis plans (i.e., advance directives jointly composed by the patient and psychiatric professionals) are important (12). Advance care planning is more complex than an advance directive; it is developed as part of a continual dialogue between (mostly older and multimorbid) patients and their multiprofessional caregivers.

## Illegal and legal drugs

Illegal drugs represent another interface between psychiatry and law. Practices range from the liberal to the restrictive. The key issues include legalization (particularly regarding cannabis); substitution (opioids, substitute drugs, or heroin); in-patient and out-patient treatment orders; and criminal liability. Questions regarding the effectiveness of particular drug policies and whether drug addicts should be regarded as ill or criminal have never been definitively answered. In addition to effective prevention, the following issues need to be considered in a legal context: service accessibility, the right to receive treatment, psychosocial support, and reimbursement, particularly for addicts with complex problems (e.g., homelessness, procurement criminality, and severe health challenges). There is a conflict between the State's duty to protect people from dangerous substances that can cause severe dependency and (particularly in the case of cannabis) chronic severely impairing psychotic disorders on the one hand and the need to provide individuals with treatment and care rather than criminalizing and marginalizing them on the other.

In the case of legal drugs, public smoking bans have been instituted in a great many countries within the past decade, and these have had a (perhaps to be expected) substantial impact on public health. A special issue for psychiatry is complete smoking bans in psychiatric hospitals that raise particular ethical issues. Evidence of efficacy on abstinence and with regards to violent behavior is inconsistent. Alcohol use in the general population is strongly associated with the prevalence of alcohol-related mental and physical disorders, violence, and with the incidence of violent fatalities both on the sides of perpetrators and victims. Hence, regulating alcohol consumption through licensing, bans on drinking in the public, and restricting availability to adolescents can have a considerable impact on public health and public mental health (13).

## Delegation and substitution of professional activities

An issue of increasing importance in most countries has its origins in the difficulties relating to labor markets for psychiatric professionals. The question of “who is allowed to do what?” in mental health care appears simple enough, but the answer is rarely so. Psychiatrists and qualified nurses (along with resources) are in short supply in many countries, and many posts lay unfilled as professionals commonly migrate to work in health care systems they are not familiar with. Enduring language problems are frequent and probably are more an issue in psychiatry and psychotherapy than in more technical medical specialties. Nonetheless, requirements of quality of treatment have increasingly been specified in law, especially in high-income countries. How to manage the widening gap between such legal demands and the availability of qualified specialists is a pressing issue. In many countries, future legislators will have to define more clearly which tasks can be delegated to which professional groups or substituted by other professions.

## Conclusion

We have provided a brief overview of the numerous interfaces between psychiatry and law, each of which can have a considerable impact on clinical practice. Ideas of patient autonomy and patients' rights have led to manifold changes in these interfaces. Legal frameworks influence clinical practice and shifting psychiatric and ethical concerns make their way into the public consciousness and onto the statute books. Such processes are dynamic, multilateral, and omnidirectional. What is more, they differ both between and within countries. Especially given the extent of change over the past two decades, there is every reason to believe that the relationship between psychiatry and the law will continue to evolve; our current ethical views and legal frameworks are no more than momentary judgements on our societies and health care systems.

## Data availability statement

The original contributions presented in the study are included in the article/supplementary material, further inquiries can be directed to the corresponding author/s.

## Author contributions

All authors listed have made a substantial, direct, and intellectual contribution to the work and approved it for publication.

## Conflict of interest

The authors declare that the research was conducted in the absence of any commercial or financial relationships that could be construed as a potential conflict of interest.

## Publisher's note

All claims expressed in this article are solely those of the authors and do not necessarily represent those of their affiliated organizations, or those of the publisher, the editors and the reviewers. Any product that may be evaluated in this article, or claim that may be made by its manufacturer, is not guaranteed or endorsed by the publisher.
